# Detection of Interfacial Debonding in a Rubber–Steel-Layered Structure Using Active Sensing Enabled by Embedded Piezoceramic Transducers

**DOI:** 10.3390/s17092001

**Published:** 2017-09-01

**Authors:** Qian Feng, Qingzhao Kong, Jian Jiang, Yabin Liang, Gangbing Song

**Affiliations:** 1 Key Laboratory of Earthquake Geodesy, Institute of Seismology, China Earthquake Administration, Wuhan 430071, China; fengqian@eqhb.gov.vn (Q.F.); jiangjian@eqhb.gov.cn (J.J.); yabinliang@eqhb.gov.cn (Y.L.); 2 Wuhan Institute of Earthquake Engineering Co. Ltd., Wuhan 430071, China; 3 Department of Mechanical Engineering, University of Houston, 4800 Calhoun, Houston, TX 77204, USA; qkong@uh.edu

**Keywords:** interfacial debonding, rubber–steel-layered structures, piezoceramic transducers, active sensing

## Abstract

Rubber–steel-layered structures are used in many engineering applications. Laminated rubber–steel bearing, as a type of seismic isolation device, is one of the most important applications of the rubber–steel-layered structures. Interfacial debonding in rubber–steel-layered structures is a typical failure mode, which can severely reduce their load-bearing capacity. In this paper, the authors developed a simple but effective active sensing approach using embedded piezoceramic transducers to provide an in-situ detection of the interfacial debonding between the rubber layers and steel plates. A sandwiched rubber–steel-layered specimen, consisting of one rubber layer and two steel plates, was fabricated as the test specimen. A novel installation technique, which allows the piezoceramic transducers to be fully embedded into the steel plates without changing the geometry and the surface conditions of the plates, was also developed in this research. The active sensing approach, in which designed stress waves can propagate between a pair of the embedded piezoceramic transducers (one as an actuator and the other one as a sensor), was employed to detect the steel–rubber debonding. When the rubber–steel debonding occurs, the debonded interfaces will attenuate the propagating stress wave, so that the amplitude of the received signal will decrease. The rubber–steel debonding was generated by pulling the two steel plates in opposite directions in a material-testing machine. The changes of the received signal before and after the debonding were characterized in a time domain and further quantified by using a wavelet packet-based energy index. Experiments on the healthy rubber–steel-layered specimen reveal that the piezoceramic-induced stress wave can propagate through the rubber layer. The destructive test on the specimen demonstrates that the piezoceramic-based active sensing approach can effectively detect the rubber–steel debonding failure in real time. The active sensing approach is often used in structures with “hard” materials, such as steel, concrete, and carbon fiber composites. This research lays a foundation for extending the active sensing approach to damage detection of structures involving “soft” materials, such as rubber.

## 1. Introduction

Due to its simple structure, low cost, and convenient installation, laminated rubber bearing, as one type of the elastomeric seismic isolation device, has been increasingly deployed to buildings, bridges, and nuclear structures to reduce structural motions from earthquakes [1,2,3]. During the service life of rubber bearings, delamination or debonding between the rubber layers and the steel plates is one of the typical failure modes that may reduce the damping capacity and even result in the complete bearing system failure [4,5]. However, the initial delamination often occurs at the internal rubber–steel interfaces and the detection of the internal debonding damage is very difficult. Observation of the displacement or stress curves from the compression or shear tests of the rubber bearings is the main method of determining the debonding or delamination of the seismic isolation bearing [6,7]. However, this method can be used to test the performance or characterize the mechanical properties of the rubber bearing system in the laboratory, but not suitable for the detection of the debonding in real time in practical applications.

In recent years, many methods have been proposed to monitor and detect the occurrence of debonding or delamination [8,9,10,11,12,13,14] in laminated composite structures. Grouve et al. [8] investigated the resonance frequencies of carbon-PEI laminated cantilever beams using embedded fiber Bragg grating (FBG) strain sensors, and both experimental and analytical test samples show the resonance frequencies are a function of both delamination parameters and laminate lay-up. Niell et al. [9] embedded an optical fiber inside a structural component, and the strain along the fiber can be determined when a load moves along the component, and the damage can then be detected by correlating changes in the plot with the presence of delamination. Clemente et al. [10] successfully applied the infrared thermography technique to detect the delamination-type defects for the inspection of GLAss REinforced (GLARE). Shah et al. [11] studied the delamination response of a laminated plate with piezoelectric layers and developed a quasi-3D finite-element model, which demonstrated that the presence of delamination and its growth in an interface of the laminate can be detected by changes in the electric field on the piezoelectric layer in its vicinity. Okabe et al. [12] integrated a macrofiber composite (MFC) actuator and fiber Bragg grating sensors into the composite laminates, and a new delamination detection method was proposed on the basis of the mode conversions for the multiple modes of Lamb waves, which was identified by transmitting and receiving the symmetric and antisymmetric modes separately. When antisymmetric modes were excited, the frequency dispersion of the received A_1_ mode changed depending on the delamination length owing to the mode conversion between the A_1_ mode and the S_0_ mode.

On the other hand, stress waves are often used for structural health monitoring [15,16,17,18,19]. Lead zirconate titanates (PZTs), a type of piezoceramic material, is used for stress wave generation [20,21,22,23] due to its high piezoelectric effect, low cost, fast response, wide bandwidth, and capability of actuation and sensing [24,25]. For example, Providakis et al. [26] proposed a structural health monitoring approach that integrates both electromechanical admittance (EMA) and guided wave (GW) technique by employing a piezoelectric transducer, and then successfully applied to the finite element models of damages occurring in conventional concrete specimens. With proper protection of the fragile PZT patches, PZT transducers can be also embedded into structures for structural health monitoring [27,28]. By embedding “smart aggregate” transducers and bonding piezoelectric patches in arrays at both shear spans of the beam, Chalioris et al. [29] experimentally investigates the effectiveness of an structural health monitoring technique using the admittance measurements of PZT transducer mounted on a shear-critical RC in order to detect the identify potential damage. Voutetaki et al. [30] and Chalioris et al. [31] experimentally investigated the developed wireless impedance/Admittance monitoring system in RC beams by measuring the voltage signatures of an array of embedded piezoelectric transducers as “smart aggregates.” In recent years, various applications of monitoring the interfacial damage for laminated composite structures using PZT-based transducers have been reported [32,33,34,35,36]. For concrete-encased composites structures, Qin et al. [34] and Zeng et al. [33] proposed an active sensing approach using PZT-based smart aggregates (SAs) to detect the initiation and to monitor the development of bond-slip between steel plate and concrete by investigating and comparing the stress wave propagation and attenuation, Liang et al. [36] also successfully detected the bond-slip occurrence by employing electro-mechanical impedance (EMI) technique using piezoceramic transducers. For rubber-related composite structures, piezoceramics were used to fabricate the PZT/polymer composite transducer by sandwiching one PZT/polymer middle layer and two solid PZT surface layers [37], or the piezoelectric-rubber by mixing the PZT particles into silicon rubber [38,39,40]. In addition, the smart piezoelectric materials have also emerged as a potential tool for the online aging monitoring of rubber by employing the EMI sensing technique [41]. However, to the authors’ best knowledge, no debonding detection for rubber-layered structures using the active sensing method has been reported.

This paper develops a simple but effective piezoceramic based active sensing approach to detect the debonding failure of the rubber–steel-layered structure in real time. A three-layer steel–rubber–steel sandwiched specimen was fabricated in the laboratory. For each steel plate, four pre-designed cavities were machined on one side of the plate surface so that four piezoceramic transducers can be fully embedded in each steel plate. This installation technique does not change the geometry and surface conditions of the steel plates so that the performance of the rubber bearing will not be influenced. In the active sensing approach, piezoceramic transducers embedded in one steel plate generates the designed stress wave, which can be detected by the corresponding transducers embedded in the other plate. Experimental results on the healthy steel–rubber-layered structure (without debonding) reveal that the piezoceramic induced stress wave can propagate through the rubber layer and be detected by the piezoceramic sensor. When the delamination occurs, the debonding interfaces will attenuate the stress wave so that the amplitude of the received signal will decrease. The rubber–steel delamination was generated by pulling the two steel plates in opposite directions in material testing machine. The energy loss of the stress wave before and after the delamination was observed from the received signal in time domain and further quantified in the wavelet packet-based energy index, which makes it possibility for the proposed technique to be potentially applied to seismic isolation rubber bearings.

The active sensing approach is often used in structures with “hard” materials, such as steel, concrete, and carbon fiber composites. To the authors’ best knowledge, this research might be the first study of applying active sensing to rubber, a “soft” material with a much higher damping, as compared with “hard” materials. This original research lays a foundation to extend the active sensing approach to damage detection of structures involving “soft” materials.

## 2. Principles

### 2.1. Piezoceramic-Based Active Sensing Approach

**Figure 1:**
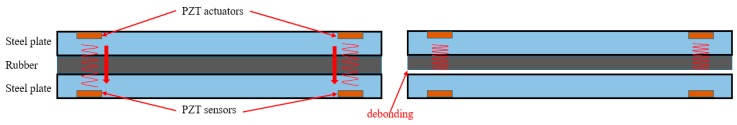
Schematic of the active sensing approach in the application of the detection of the rubber–steel bond failure.

Due to the piezoelectricity, piezoceramic transducers can be either used as actuators or sensors. In the piezoceramic-based active sensing approach, one PZT transducer is used as an actuator to generate stress wave. The propagating stress wave can be detected by the other PZT transducer (PZT sensor) which is deployed on the wave path. Figure 1 gives the schematic of the active sensing approach in the application of the detection of the rubber–steel bond failure. As shown in the figure, four PZT transducers were deployed in the pre-machined cavities of the steel plates. Two PZT actuators in one steel plate function as actuators to generate designed stress wave. It should be noted that Lamb waves generated by the PZT actuators mainly propagate along the steel plate but not through the structural interfaces. The stress waves in longitudinal mode can propagate through the rubber–steel interfaces between the PZT actuators and sensors. As demonstrated in the later “Experimental Results” section, the PZT-induced stress wave will propagate through the rubber layer. For a healthy steel–rubber-layered structure, the stress wave propagates through the interfaces between steel plates and the rubber layer and can finally be detected by the PZT sensors in the other steel plate. When the debonding failure occurs, the debonded interfaces will greatly attenuate the stress wave energy. The PZT sensors in this case will not detect the wave response from the actuators due to the high wave attenuation from the debonded interfaces.

### 2.2. Wavelet Packet-Based Energy Index (WPEI)

The wavelet packet-based energy analysis has been proved as an effective signal process method in characterizing the change of the signal energy [42,43]. Based on the wavelet decomposition, a signal can be decomposed into several wavelet packets and the signal energy of each packet can be computed. Consequently, the energy of the signal can be computed by the summation of energies of all the packets. The procedure of the computation of WPEI is given in Equations (1)–(3) as follows:

X_j_ = [X_j,1_, X_j,2_,…, X_j,m_], (1)

E_i,j_ = ||X_j_||2 = x_j,1_^2^ + x_j,2_^2^ +···x_j,m_^2^, (2)

(3) $$E=\overset{2^{n}}{\underset{k=1}{\sum }}E_{i,k}$$

where X_j_ is the decomposed signal from the original signal X; j is the frequency band (j = 1…2^n^); E_i,j_ is the energy of the decomposed signal; m is the sampling data collected from the data acquisition system; E is the computed energy, which is used to present the energy of the original signal.

The WPEI has been employed to quantitatively monitor or evaluate the structural health condition in various applications of concrete structures. In this research, the WPEI was applied to quantity the energy loss of the received signal in the active sensing approach before and after the rubber–steel bond failure.

## 3. Experimental Setup and Procedure

### 3.1. Specimen with Installed PZT Transducers

**Figure 2:**
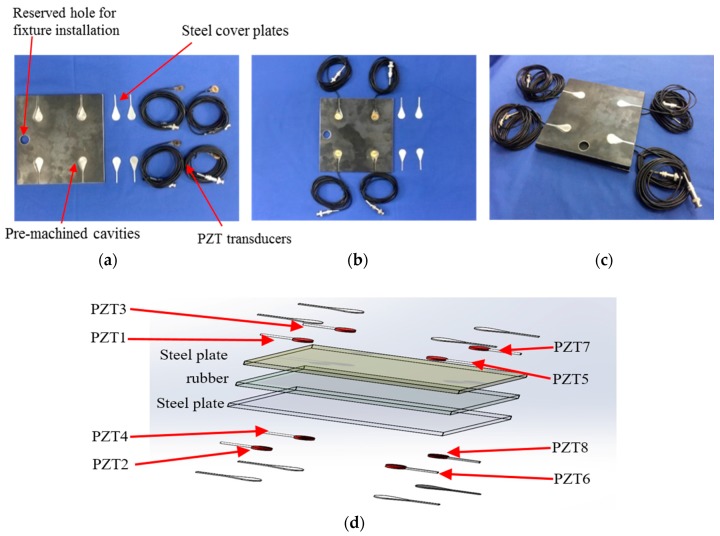
PZT transducer installation: (**a**) A steel plate with four pre-machined cavities; (**b**) PZT transducer installed in the cavities with epoxy; (**c**) the specimen with embedded PZT transducers; (**d**) schematic of the PZT transducer installation.

In this research, a three-layer steel–rubber–steel sandwiched specimen consists of one rubber layer and two steel plates bonded together with epoxy resin was fabricated in the laboratory. For each steel plate, four PZT transducers (PZT-5H) were installed in the pre-machined cavities of the steel plate, as shown in Figure 2. The detailed properties of the specimen can be found in Table 1. The installation procedures are as follows: Firstly, four PZT transducers with lead wires were installed in the four cavities of the steel plate with epoxy, as shown in Figure 2b. Secondly, four machined steel plate covers with the same geometry as the cavities were mounted on the top of the PZT transducers with epoxy. The total thickness of the epoxy layer, PZT transducer, and the steel plate cover is equal to the thickness of the cavity so that the proposed transducer installation method does not change the geometry and the surface conditions of the steel plate. The steel–rubber–steel sandwiched specimen with embedded PZT transducers is shown in Figure 2c, and the 3D PZT transducer installation schematic is shown in Figure 2d.

### 3.2. Experimental Setup and Procedure

**Figure 3:**
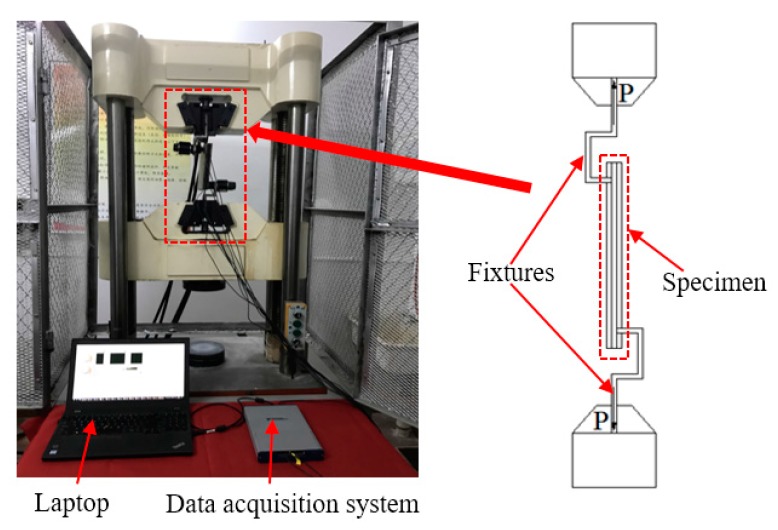
Experimental setup.

**Figure 4:**
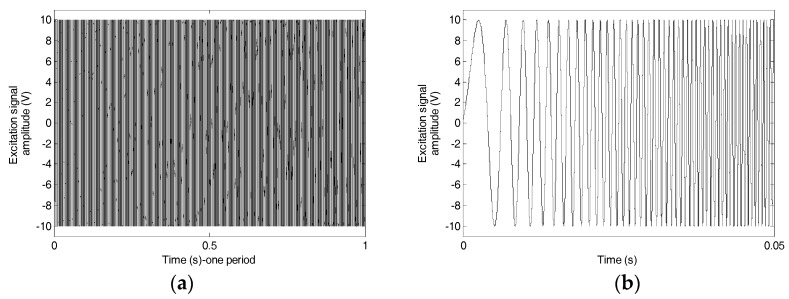
Excitation signal: (**a**) one period of the excitation signal; (**b**) one section of one period of the excitation signal.

The experimental setup, including the test specimen, the loading machine, a data acquisition system (NI-USB 6363, National Instruments, Austin, TX, USA) with a laptop, is shown in Figure 3. In the loading test, the top and bottom fixtures were fixed in the reserved hole of the two steel plates. The rubber–steel debonding was generated by pulling the bottom steel plate downward with a tensile load rate of 0.1 kN/s using the loading machine. During the loading test, PZT1, PZT3, PZT5, and PZT7 from one of the steel plates were used as actuators. PZT2, PZT4, PZT6, and PZT8 were utilized as sensors to detect the wave response. The actuating signal of each PZT actuator was a swept sine wave signal with the amplitude, the frequency range, and the period of 10 V, 100 Hz–150 kHz, and 1s, respectively. Figure 4 displays a plot of the excitation signal.

## 4. Experimental Results

### 4.1. Loading Results

**Figure 5:**
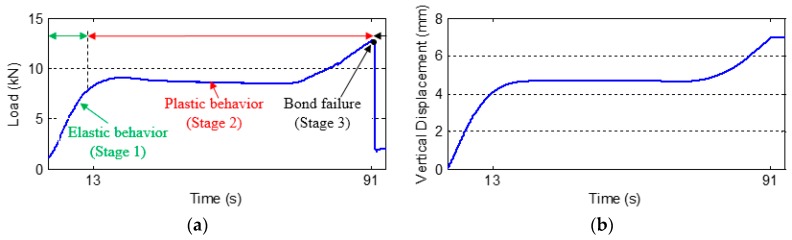
Loading results. (**a**) Load vs. Time. (**b**) Displacement vs. Time.

Figure 5 shows the both the force and displacement time histories, which clearly reveal three stages. The rubber exhibited elastic behavior in the first stage (Stage 1) and plastic behavior in the second stage (Stage 2). The rubber–steel bond failure (debonding) (Stage 3) occurred at around 91 s in the loading test. After the time of failure, the load suddenly dropped due to the debonding at the interfaces between the steel plates and the rubber. Due to the full protection of the embedded PZT transducers by the steel cover plates, all the PZT transducers were not damaged and the functionality of the PZT transducers maintain during the whole test.

### 4.2. Time Histories of the Received Signals

**Figure 6:**
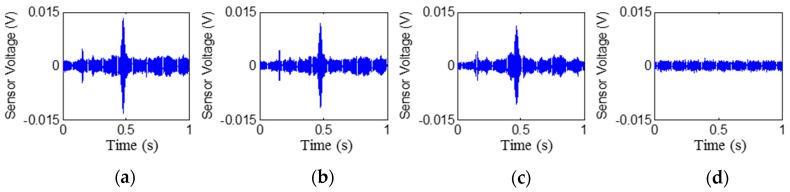
Signal received by PZT 2 (PZT1–PZT2 pair): Stage 0 to Stage 3 from left to the right. (**a**) Stage 0 ; (**b**) Stage 1; (**c**) Stage 2; (**d**) Stage 3.

**Figure 7:**
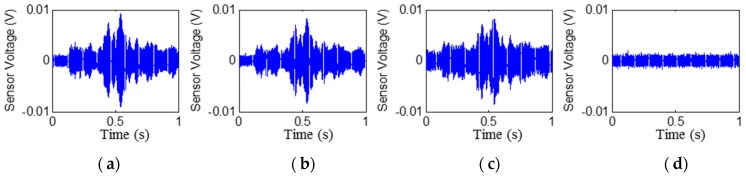
Signal received by PZT 4 (PZT3–PZT4 pair): Stage 0 to Stage 3 from left to the right. (**a**) Stage 0; (**b**) Stage 1; (**c**) Stage 2; (**d**) Stage 3.

**Figure 8:**
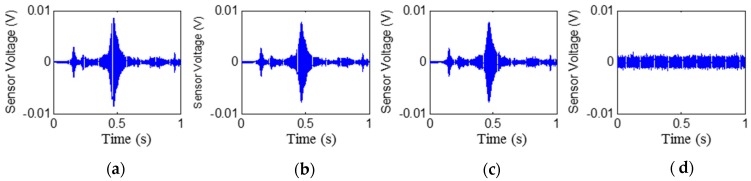
Signal received by PZT 6 (PZT5–PZT6 pair): Stage 0 to Stage 3 from left to the right. (**a**) Stage 0; (**b**) Stage 1; (**c**) Stage 2; (**d**) Stage 3.

**Figure 9:**
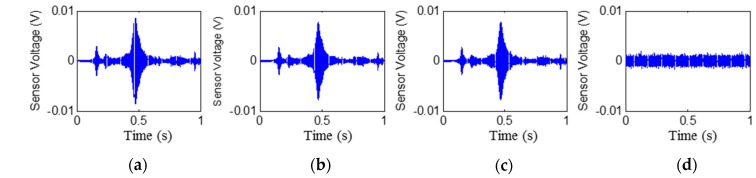
Signal received by PZT 8 (PZT7–PZT8 pair): Stage 0 to Stage 3 from left to the right. (**a**) Stage 0; (**b**) Stage 1; (**c**) Stage 2; (**d**) Stage 3.

The time histories of the signals received by PZT2, PZT4, PZT6, and PZT8 are shown in Figure 6, Figure 7, Figure 8 and Figure 9, respectively. Stage 0 represents the initial received signal (healthy status) that was measured before the loading test. It is clear that the PZT-induced stress wave can propagate through the rubber layer when the steel–rubber-layered structure is in its health status (without debonding). Due to the high damping properties, the stress wave propagation is subject to more attenuation in the rubber material. Therefore, the amplitude of the received signal from sensors was lower (close to 0.01V) than other cases when the stress wave propagates through concrete or steel. On the other hand, the rubber–steel-layered structure has multiple interfaces of rubber and steel. The complex interfacial condition will also attenuate the wave propagation energy. During the loading process, the received signal of each PZT sensor from Stage 1 to Stage 2 did not change much; therefore, only one received signal sample from each stage is presented and is shown in the figure. The received signal from Stage 3 presented in the figure was measured one second after the debonding event in the loading test. It can be seen that, for each PZT sensor, the received signal in the time domain does not change much from Stage 0 to Stage 2. When the rubber–steel debonding occurred, the amplitude of the received signal for all the PZT sensors suddenly dropped to near zero due to the high wave attenuation of the debonded interfaces between the steel plates and the rubber layer. Destructive experimental results of the received signal from the time domain show that the piezoceramic-based active sensing approach is capable of detecting the rubber–steel debonding failure in real time.

### 4.3. Wavelet Packet-Based Energy Index

**Figure 10:**
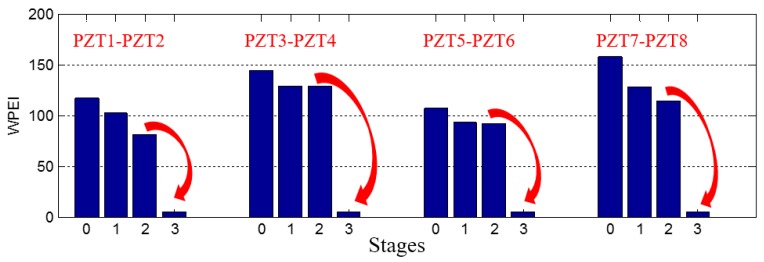
Wavelet packet-based energy indices.

To quantify the energy of the received signal, the wavelet-packet based energy index was applied, as shown in Figure 10. From the energy indices, the sudden wave energy loss due to the debonding can be directly observed at Stage 3 for all the PZT sensors. In addition to the detection of the debonding, a downward trend of the received signal energy of all the PZT sensors can be found from Stage 0 to Stage 2. Though debonding initiation was not clearly presented in the WPEI, the proposed method shows the possibility to be an in-situ or real time method to detect the debonding event. One possible reason is the deterioration of the bonded interfaces between the steel plates and the rubber layer during the loading test. In addition to the detection of the debonding, the wavelet packet-based energy index may have the potential to detect the deterioration of the bonding condition of the rubber–steel interfaces.

## 5. Conclusions

In this paper, a simple but effective piezoceramic-based active sensing approach was developed to detect the rubber–steel debonding of a laminated rubber–steel structure in real time. The proposed transducer installation technique can ensure the functionality of PZT transducers without changing the geometry and surface conditions of the steel plates. Experiments on the healthy rubber–steel-layered specimen reveal that the piezoceramic-induced stress wave can propagate through the rubber layer. The destructive testing results show that both the received signal in the time domain and the wavelet packet-based energy index are capable of detecting the rubber–steel debonding failure in real time. In the authors’ future work, more tests will be conducted to verify the reliability of the proposed method and the possibility for the detection of the debonding initiation at the early age. The feasibility of implementing the current transducer installation method to seismic isolation rubber bearings with remote monitoring technology will be explored [44,45]. In addition, the relationship between the responses of the PZT transducers and the debonded surfaces morphology will be investigated.

The active sensing approach is often used in structures with “hard” materials, such as steel, concrete, and carbon fiber composites. To the authors’ best knowledge, this research might be the first study in which active sensing is applied to rubber, a “soft” material with a much higher damping, as compared with “hard” materials. This research lays a foundation to extend the active sensing approach to damage detection of structures involving layered “soft” materials. Such structures include a pipeline with inner rubber lining, a metal shaft with integrated rubber seal, and an elastomer vibration isolator bonded on a metal surface.

## Figures and Tables

**Table 1:** Properties for the test specimen.

Materials	Parameters	Values	Unit
Steel plate	Dimension	20 × 20 × 0.6	cm
	Young’s modulus	206	GPa
	Density	7.9	g/cm^3^
	Poisson’s ratio	0.25	-
Rubber layer	Dimension	20 × 20 × 0.5	cm
	Young’s modulus	0.0078	GPa
	Density	1.27	g/cm^3^
	Poisson’s ratio	0.47	-
PZT-transducer	Dimension	φ18 × 3	mm
	Piezoelectric strain coefficients	2450	pC/N
